# OMICS IN AGING RESEARCH: FROM BIOMARKERS TO SYSTEMS BIOLOGY

**DOI:** 10.1093/geroni/igz038.869

**Published:** 2019-11-08

**Authors:** Daniel Promislow

**Affiliations:** University of Washington, Seattle, Washington, United States

## Abstract

Advances in whole genome sequencing have dramatically increased our potential to understand what shapes variation in rates of aging and age-related disease in natural populations, but we are still far from realizing this potential. Researchers have identified thousands of genetic markers associated with complex human traits. However, these markers typically explain a very small fraction of the observed variance, leaving an enormous explanatory gap between genotype and phenotype. I will present data from diverse species to illustrate the power of so-called endophenotypes—the epigenome, transcriptome, proteome, and metabolome—to bridge the genotype-phenotype gap. Using multivariate and network models that integrate genetic information with other endophenotype variation, we are closer than ever to understanding the mechanisms that account for natural variation in aging and age-related disease, and the evolutionary forces that have shaped that variation.

